# Development and Performance Evaluation of a Bevameter for Measuring Soil Strength

**DOI:** 10.3390/s21041541

**Published:** 2021-02-23

**Authors:** Ji-Tae Kim, Dong-U Im, Hyuek-Jin Choi, Jae-Won Oh, Young-Jun Park

**Affiliations:** 1 Department of Biosystems Engineering, Seoul National University, 1 Gwanak-ro, Gwanak-gu, Seoul 08826, Korea; jitaekim10@snu.ac.kr (J.-T.K.); ehddn0112@snu.ac.kr (D.-U.I.); 2 Global Smart Farm Convergence Major, Seoul National University, 1 Gwanak-ro, Gwanak-gu, Seoul 08826, Korea; 3 Offshore Industries R&BD Center, Korea Research Institute of Ships and Ocean Engineering, 1350 Geojebuk-ro, Geoje, Gyeongsangnam-do 53201, Korea; hjchoi@kriso.re.kr (H.-J.C.); herotaker@kriso.re.kr (J.-W.O.); 4 Research Institute of Agriculture and Life Science, Seoul National University, 1 Gwanak-ro, Gwanak-gu, Seoul 08826, Korea

**Keywords:** bevameter, soil strength, slip sinkage, pressure-sinkage relationship, shear stress-slip displacement relationship

## Abstract

The driving performance of an off-road vehicle is closely related to soil strength. A bevameter is used to measure the soil strength, and it usually consists of two independent devices: a pressure–sinkage test device and a shear test device. However, its development and measurement processes have not been standardized; thus, researchers apply it in various fields according to their own discretion. In this study, a new bevameter was developed, and experiments were conducted to clarify the factors that affect the measurement performance of the bevameter. The pressure–sinkage test device was tested with circular plates of different sizes, and the results confirmed that the pressure–sinkage parameters decreased with the plate size. For the shear-test device, normal pressure was applied using a dead load to prevent normal-pressure variation due to displacement and speed. In addition, a spline was installed on top of the shaft connected to the shear ring to measure slip sinkage during the shear test. The results showed that the slip sinkage increased in proportion to the normal pressure and slip displacement, but the increase gradually decreased and converged to a certain point.

## 1. Introduction

The driving performance of a vehicle is determined by its driving force, which is determined by the engine and strength of the surface on which the vehicle traverses. For vehicles driving on roads, the thrust is determined by the engine of the vehicle, and the road surface provides sufficient strength. For off-road vehicles (e.g., military vehicles, agricultural vehicles, construction equipment, and planetary exploration rovers), the thrust is determined by the strength of the soil, which is generally in a natural state and does not offer sufficient strength. Therefore, understanding the soil strength and its interaction with the driving vehicle is essential for designing off-road vehicles and predicting their performance. The study of the relationship between the performance of off-road vehicles and the soil strength is called terramechanics or vehicle traction mechanics.

Approaches used to study the dynamics of driving devices and soil can generally be divided into experimental and analytical approaches. Experimental methods focus on determining whether the strength of the soil can allow the driving device to produce sufficient momentum while supporting the target vehicle. During World War II, the US Army Waterways Expert Station (WES) developed a cone penetrometer that was used to define the mechanical properties of soil according to a cone index. The cone penetrometer experimentally measures the mechanical properties of soil to predict whether it can support a military vehicle. The shape, size, and test method of a cone penetrometer are specified in ASAE S313.3:1999(R2013) [1]. The cone penetrometer is small and lightweight; thus, it is convenient to use onsite. However, the measurement values change depending on the operator’s proficiency. In addition, the cone index denotes the relationship between the compression and shear properties; these parameters cannot be separated. Thus, it is inapplicable to the design of driving devices for off-road vehicles, and it has limited applicability for predicting the performance of driving device on new terrain [2].

The bevameter was developed to compensate for the shortcomings of cone penetrometers and to study the interrelationship between the driving device of a vehicle and the supporting soil. A bevameter is used for measuring the mechanical properties of soil, and it was first presented by Bekker [3]. A bevameter consists of a pressure–sinkage test device and shear test device. It was developed to measure the mechanical properties of soil by applying load conditions similar to those applied in the case of a driving device with two separate devices. An off-road vehicle applies vertical and shear loads on the soil. The pressure–sinkage test device measures the mechanical properties of soil in the vertical direction based on the correlation between the sinkage and pressure generated by a compressive load. The shear test device measures the horizontal mechanical properties through the correlation between the slip displacement and the shear stress generated by a shear load. In experimental approaches, the cone penetrometer is used to predict the operability of a specific driving device. In analytical approaches, the bevameter is used to predict the driving performance of a driving device. The bevameter most closely represents the interaction between a driving device and soil, but the onsite measurement process is more complex and time-consuming than that of a cone penetrometer [2,4]. Thus far, it is still used in research on terramechanics.

To compensate for the shortcomings of bevameter, Wills [5] developed a semiautomatic bevameter where hydraulic pressure is used to apply an axial load and torque; this allows a single researcher to conduct research with a bevameter. Golob [6] developed a bevameter that can perform both pressure–sinkage and shear tests with a single hydraulic cylinder and that could store digital data rather than analog data. Wong and Preston-Thomas [7] developed a bevameter that could be attached to a tractor, which they used to test the mechanical properties of soil covered by snow. They derived an equation for soil that exhibits a hump during the shear test. Okello et al. [8] confirmed that the traction predicted from the mechanical properties of soil [3] reasonably match the traction measured in actual tests. Park and Lee [9] developed an excavator-mounted bevameter to measure the mechanical properties of soil and predict the traction performance of crawler-type vehicles. Bodin [10,11] developed a bevameter to measure the mechanical properties of snow and to research military vehicle. Plessis and Yu [12] used the pressure–sinkage parameter proposed by Bekker [3] to predict the normal pressure of a vehicle and confirmed similar behavior to that of the actual normal pressure. Massah and Noorolahi [13] developed a tractor-attached bevameter to measure the mechanical properties of soil with circular, elliptical, and rectangular plates; they confirmed that the shape of the plate affects the measurements. Apfelbeck et al. [14] varied the height, number, and shear speed of the grouser to identify factors that affect the shear test; their results showed that the shear stress increases with the height of the grouser. They confirmed that the number of grousers and shear speed did not affect the shear stress. Edwards et al. [15] used a bevameter to measure the mechanical properties of the simulant Fillite (grade 500W-LF) in a test bed for evaluating the drivability of a planetary rover. Mahonen et al. [16] developed a portable bevameter to measure the mechanical properties of snow; they confirmed that the bevameter can be applied to snow.

The above literature review shows that bevameters have been used for research in various fields. However, in the absence of relevant regulations, bevameters have been produced and tested at the discretion of researchers. For example, the size and shape of the plate in the pressure-sinkage test and the size of the shear ring in the shear test were different in different studies. Reece [17] reported that a driving vehicle can experience sinkage caused by the vertical load and slip sinkage caused by the horizontal load. Therefore, both sinkage and slip sinkage should be considered for the motion resistance of a vehicle caused by soil subsidence. However, previously developed bevameters cannot measure slip sinkage due to shear; thus, they may underestimate the sinkage of a vehicle.

The mechanical properties of soil are measured with a cone penetrometer and bevameter to predict the thrust, motion resistance, and drivability of a vehicle and evaluate the capability of the vehicle for transport, movement, and operation. Unlike the case for the cone penetrometer, the fabrication and testing methods for the bevameter have not been standardized; thus, it is designed and studied according to researchers’ experience and discretion. In this study, a new bevameter was developed, and experiments were performed to clarify the factors that affect the measurements of the component devices. For the pressure–sinkage test device, different plate sizes were used to evaluate their effect on the measurements. For the shear-test device, instead of hydraulic pressure, normal pressure was applied using a dead load. Hydraulic pressure depends on the displacement and speed. If sinkage occurs during the shear test, there is a possibility that the normal pressure will vary; however, for a dead load, the pressure does not vary, even if the shear test causes sinkage because it is independent of the displacement and speed. Moreover, the weight of the vehicle affecting the soil thrust is determined by the mass of the vehicle independent of the displacement and speed. Therefore, if a dead load is used instead of hydraulic pressure, the interaction between the vehicle and soil can be more accurately simulated.

In addition, a spline was installed between the worm gear and shaft such that the shaft had a degree of freedom in the rotation and axial directions. Therefore, the normal pressure and torque were separated, enabling the sinkage occurring during the shear test to be considered.

## 2. Materials and Methods

**Figure 1:**
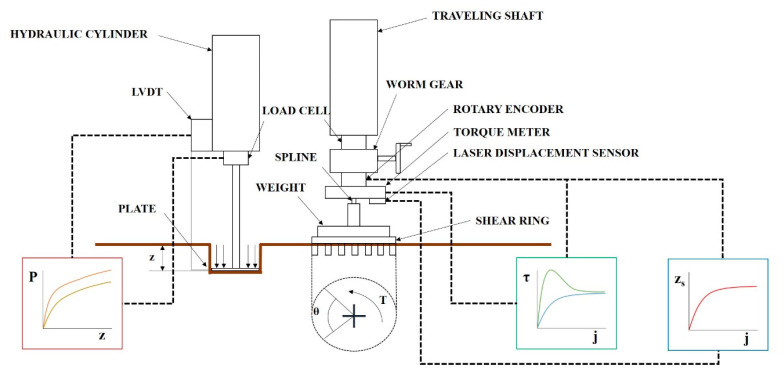
Schematic of the bevameter developed in this study.

In this study, a bevameter was developed as an attachment to a tractor. It receives hydraulic pressure from the hydraulic unit of the tractor during the pressure–sinkage test. The bevameter conforms to the category 2 three-point hitch standard for attachment to 30–75 kW tractors [18]. Figure 1 shows a schematic of the developed bevameter.

The pressure–sinkage test device measures the normal pressure of the soil by sinking plates of different sizes into the soil surface. The piston connected to the hydraulic cylinder is lowered by hydraulic pressure supplied from the tractor. When the plate connected to one end of the piston sinks into the soil, the load acting on the plate is measured by a load cell, and the sinkage of the plate is measured with a linear variable differential transformer (LVDT). The measured load is converted into pressure according to the area of the plate, and the pressure–sinkage parameter of the soil can be derived from the soil pressure–sinkage characteristic equation proposed by Bekker [3] and by using the converted pressure and measured sinkage. Bekker’s equation is as follows:

(1) $$p=\left(\frac{K_{c}}{b}+K_{\emptyset }\right)z^{n}$$

where *p* is the pressure applied to the plate (kPa), *b* is the small width or radius (m), *z* is the sinkage (m), and $K_{c}$ ($\mathrm{kN}/m^{n+1}$), $K_{\emptyset }$ ($\mathrm{kN}/m^{n+2}$), and *n* (dimensionless) are pressure–sinkage parameters.

**Figure 2:**
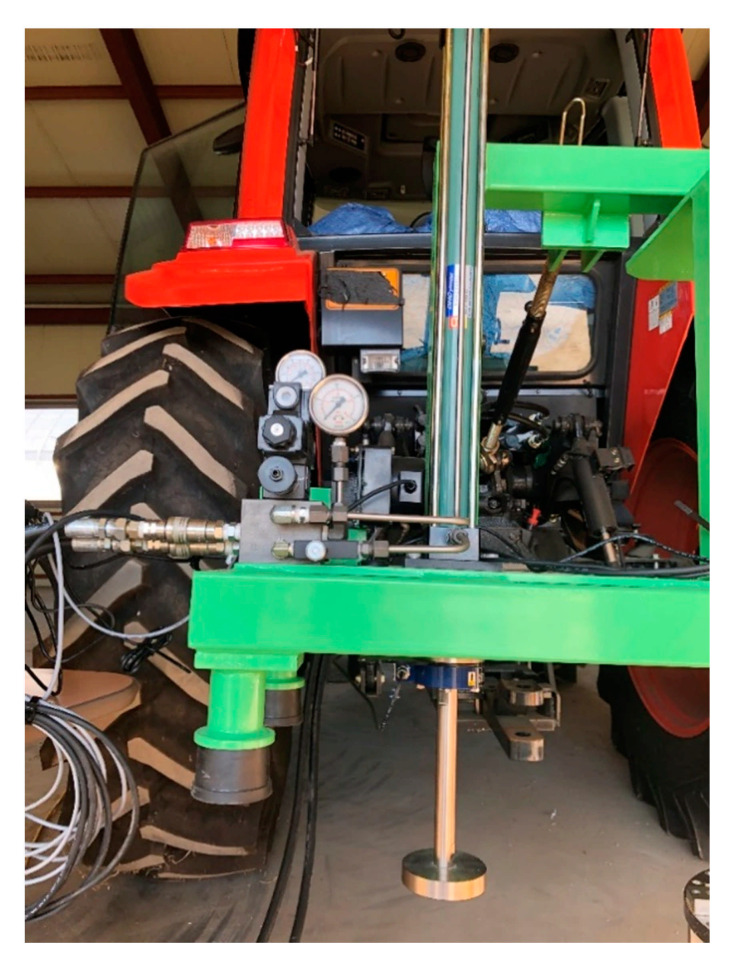
Pressure–sinkage test device of the developed bevameter.

Figure 2 shows the pressure–sinkage test device developed in this study. All the plates in this study were circular with diameters of 40, 60, 80, and 100 mm, respectively. In the test, the four pairs of plate couples were used—60–40 mm, 60–80 mm, 60–100 mm, and 100–80 mm—based on the 60 mm and 100 mm couples which were most commonly used in the current study.

The three pressure–sinkage parameters ($K_{c}$, $K_{\emptyset }$, *n*) of the soil were derived as follows, where *i* = 1, 2:

$$p_{i}=\left(\frac{K_{c}}{b_{i}}+K_{\emptyset }\right)z_{i}{}^{n_{i}}$$

(1) The log-log scale is applied to the two measured pressure–sinkage data: $K_{eq,\;i}$ = $\left(\frac{K_{c}}{b_{i}}+K_{\emptyset }\right).$
  $$\log p_{i}=\log\left(\frac{K_{c}}{b_{i}}+K_{\emptyset }\right)+n_{i}\cdot \log z_{i}\;\log p_{i}=\log K_{eq,\;i}+n_{i}\cdot \log z_{i}$$
(2) The two graphs are expressed in linear form using the least-squares method.
(3) n is derived as the average value of the slopes of the two straight lines.
(4) $K_{eq,\;i}$ is derived using the y-intercept values of the straight lines.
(5) $K_{c}$ and $K_{\emptyset }$ can be determined by the following equations: $K_{eq,\;i}$.
  $$K_{eq,\;1}=\left(\frac{K_{c}}{b_{1}}+K_{\emptyset }\right)\;K_{eq,\;2}=\left(\frac{K_{c}}{b_{2}}+K_{\emptyset }\right)$$

**Figure 3:**
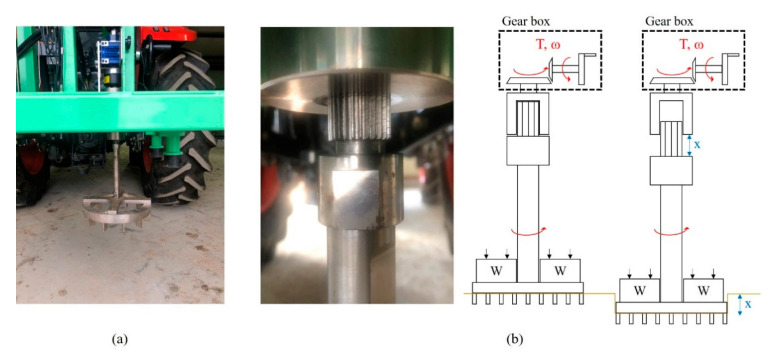
(**a**) Shear test device and (**b**) installed spline in the shear test device and schematic view.

The shear test device measures the shear stress of the soil according to slip displacement while the shear ring is rotated at a constant angular speed and a constant normal pressure is applied to the shear ring. The developed shear test device was designed to rotate the shear ring with a worm gear and hand wheel at a gear ratio of 30:1. An electric actuator was not used because that would make the initial torque difficult to control, and it was difficult to maintain a constant rotational speed when slip sinkage occurred. Figure 3a shows the shear test device. It was developed to make slip sinkage possible according to the angular displacement from a spline installed on top of the shaft. The worm gear is rotated by the hand wheel to rotate the shaft connected to the shear ring. As the shear ring rotates, the angular displacement and torque are measured with a rotary encoder attached to the shear ring and torque meter. The measured angular displacement and torque are calculated from the slip displacement and shear stress according to the dimensions of the shear ring. The slip sinkage is measured with the shear test device by a laser displacement sensor. The measured shear stress–slip displacement is used to derive the shear stress parameters, and the shear stress and vehicle parameters are used to predict the propulsion of a vehicle on the soil surface. The measured slip sinkage and slip displacement are used to predict the additional sinkage and motion resistance of a vehicle on the soil surface. Figure 3b shows the spline attached to the shear test device. 

**Figure 4:**
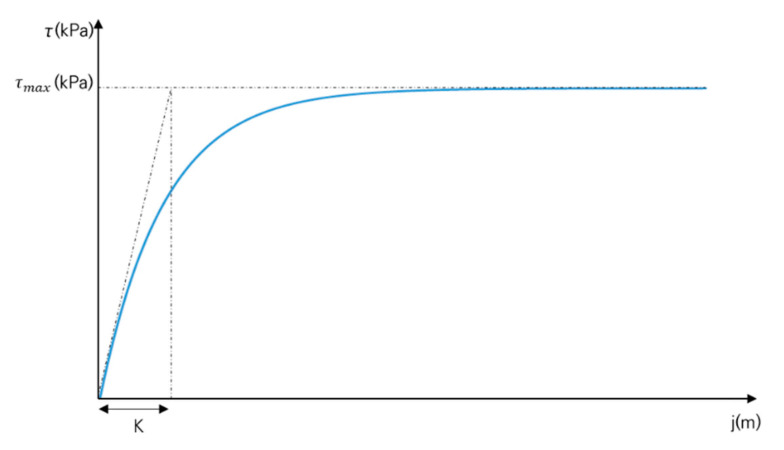
Shear stress–slip displacement relationship for soil hardening behavior.

The measured slip displacements and shear stress parameters were derived from the shear stress–slip displacement characteristic equation of Janosi and Hanamoto [19] for soil hardening behavior and the shear stress–slip displacement characteristic equation of Wong [20] for soil softening behavior. The shear stress–slip displacement characteristic equation of Janosi and Hanamoto [19] is as follows:

(2) $$\tau =\left(c+\mathrm{ptan}\emptyset \right)\left(1-e^{\frac{-j}{K}}\right)$$

where τ is the shear stress (kPa), p is the normal pressure (kPa), j is the slip displacement (m), c is the cohesion (kPa), ∅ is the angle of internal friction (°), and K is the soil deformation coefficient (m). Figure 4 shows the characteristics of soil hardening behavior. In tests with soil hardening behavior, c and ∅ can be derived with the Mohr–Coulomb failure criterion, and K can be derived from the initial slope and maximum shear stress of the measurement data [19].

**Figure 5:**
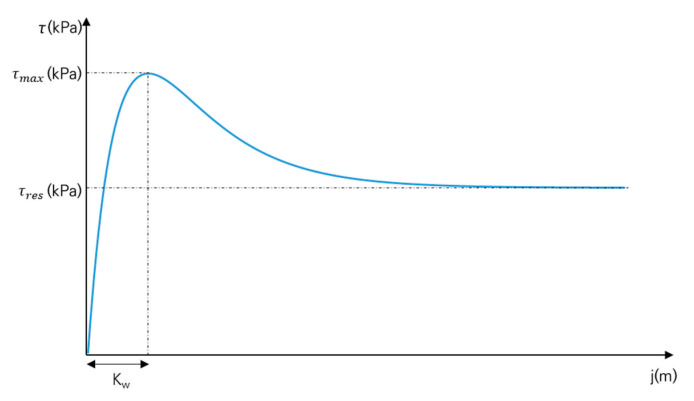
Shear stress–slip displacement relationship for soil softening behavior.

The shear stress–slip displacement characteristics equation of Wong [20] is expressed as follows:

(3) $$\tau =\left(c+\mathrm{ptan}\emptyset \right)K_{r}\left[1+\left\{\frac{1}{\left(K_{r}\left(1-\frac{1}{e}\right)\right)}-1\right\}e^{1-\frac{j}{K_{w}}}\right]\left(1-e^{\frac{-j}{K_{w}}}\right)$$

where $K_{r}$ is the ratio of the residual shear stress to the maximum shear stress (dimensionless) and $K_{w}$ is the shear deformation coefficient (m). Figure 5 shows the characteristics of soil softening behavior. In tests with soil softening behavior, c and ∅ can be derived with the Mohr–Coulomb failure criterion, $K_{r}$ can be derived from the ratio of the residual shear stress to the maximum shear stress, and $K_{w}$ can be derived from the slip displacement of the maximum shear stress. $\tau_{\max}$ refers to the maximum shear stress measured during the shear test with soil softening behavior, and $\tau_{\mathrm{res}}$ refers to the value of the shear stress that is converged to after $\tau_{\max}$.

**Figure 6:**
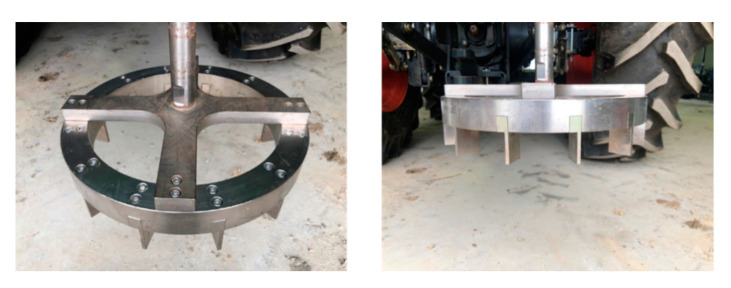
Shear ring with grousers placed at intervals of 30°.

**Figure 7:**
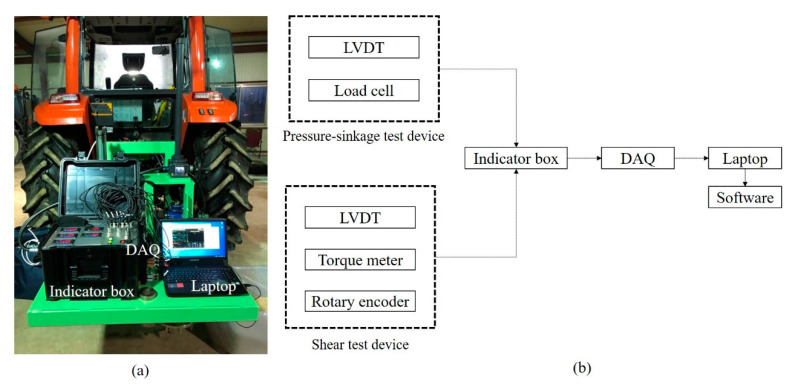
(**a**) Bevameter connected to a tractor and (**b**) block diagram of the bevameter system.

The outer and inner diameters of the shear ring developed in this study were 340 and 270 mm, respectively, and 40-mm-high grousers were placed at intervals and tightened with bolts. Figure 6 shows a shear ring with grousers placed at intervals of 30°. Figure 7 shows a bevameter attached to the three-point hitch and hydraulic port of the tractor, along with the data acquisition device (DAQ), sensors, and laptop. Table 1 summarizes the types and models of sensors used in the developed bevameter.

## 3. Results

**Figure 8:**
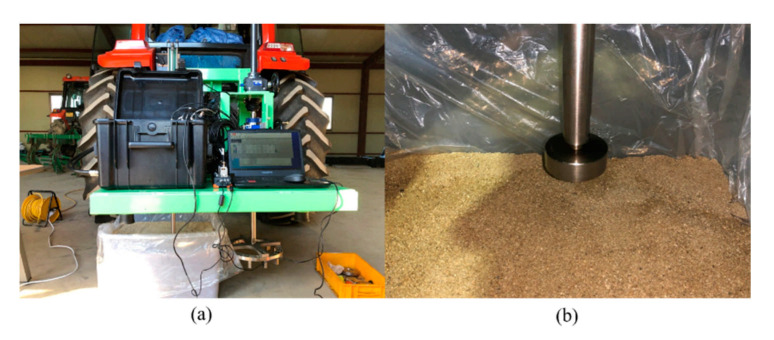
Pressure–sinkage test in the sample soil: (**a**) setup and (**b**) test in progress.

**Figure 9:**
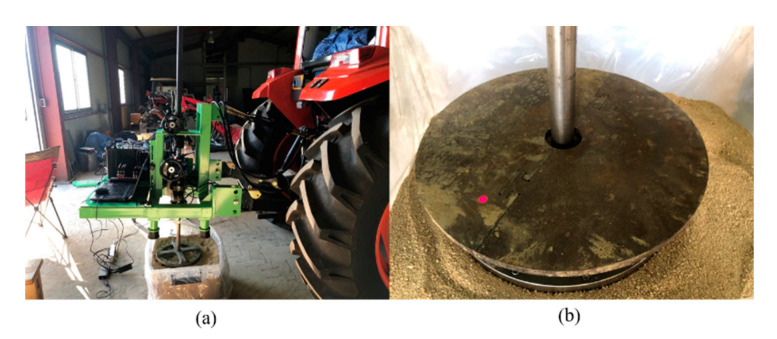
Shear test with the sample soil: (**a**) setup and (**b**) test in progress.

A pressure–sinkage test and shear test were conducted to verify the developed bevameter. Sample soil was placed in a box with dimensions of 705 × 520 × 435 mm. Figure 8 and Figure 9 show the preparation and progress of the pressure–sinkage test and shear test. To maintain the total soil quantity used in the test, soil was filled only for the first time. In addition, vinyl was placed within the test box to prevent soil loss and changes in the moisture content during the test; the soil attached to the equipment (plate, shear ring, grouser, etc.) was removed at the end of the test.

**Figure 10:**
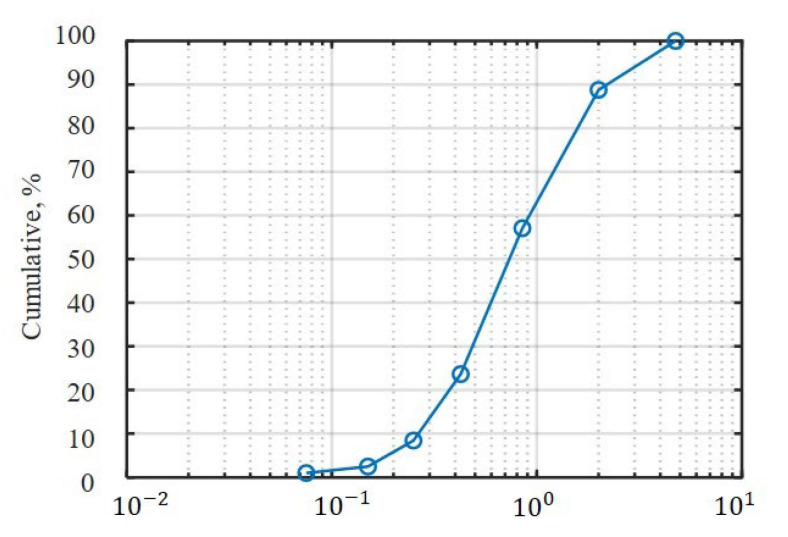
Grain-size distribution curve of the sample soil.

The physical properties of the sample soil were measured at National Instrumentation Center for Environmental Management (NICEM) of Seoul National University’s College of Agriculture and Life Sciences. Sieve analysis was conducted to analyze the soil texture using 4.75, 2, 0.85, 0.425, 0.25, 0.15 and 0.075 mm sieves. Figure 10 shows the grain-size distribution curve of the sample soil. The moisture content was measured according to the oven-drying method with a scale (BCE224I-1SKR, Sartorius, Goettingen, Germany) and dryer (CO-81, company, Seoul, South Korea). The bulk density was measured according to the methods described by Sparks et al. [21]. Table 2 lists the measurements for the soil texture, moisture content, and bulk density.

### 3.1. Pressure–Sinkage Test

Bekker [3] recommended a plate size of at least 50 mm and less than 100 mm (circular plate = radius; rectangular plate = small width) for pressure–sinkage tests in nonhomogeneous soil, and Wong [22] reported that using a circular plate rather than a rectangle or ellipse helps equalize the pressure under the plate. Thus, most studies on pressure–sinkage tests typically couple circular plates with diameters of 60 and 100 mm. In this study, four circular plates were produced with a maximum diameter of 100 mm. Four sets of tests were conducted to determine how the pressure–sinkage parameters change with the plate size. 

**Figure 11:**
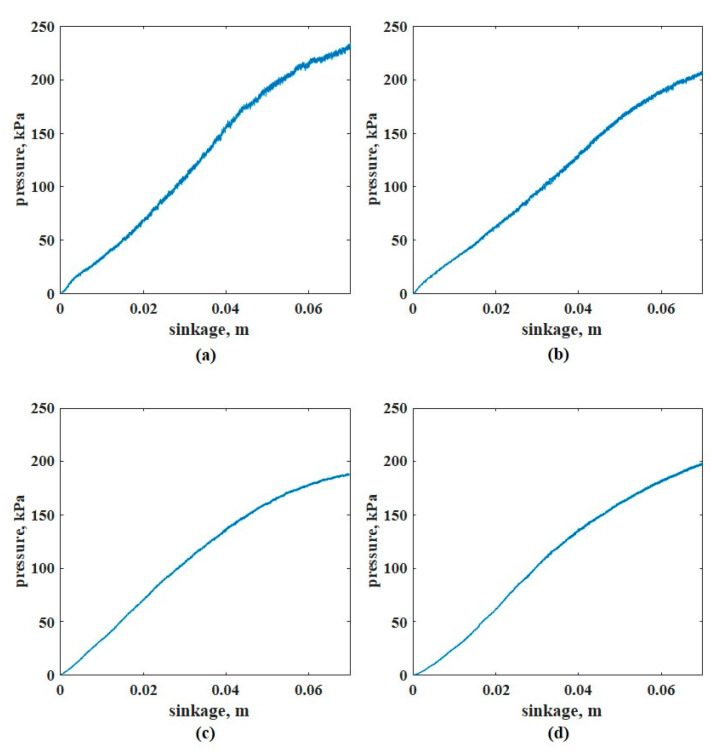
Pressure–sinkage curves with different sinkage plate diameters: (**a**) 40, (**b**) 60, (**c**) 80, and (**d**) 100 mm.

**Figure 12:**
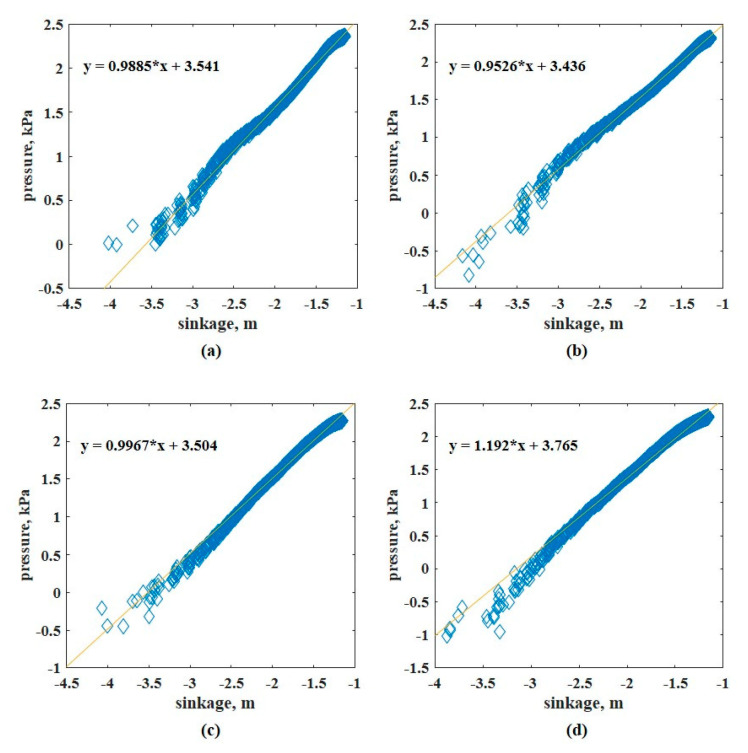
Pressure–sinkage curves of Figure 11 on a log-log scale: (**a**) 40 (**b**) 60, (**c**) 80, and (**d**) 100 mm.

A penetration velocity of 2.11 mm/s was applied for all the pressure-sinkage tests. In Case #1, the plates were set to the commonly used sizes in previous studies. Figure 11 shows the pressure–sinkage relationship in each test. To derive the pressure–sinkage parameters, the log-log scale was applied to the measured pressure–sinkage data as previously mentioned (Figure 12); the pressure-sinkage parameter was derived using Equation (1). Table 3 lists the derived pressure–sinkage parameters.

The pressure-sinakge parameter was shown differently in Case #1~4, which means that size of pressure plate affects pressure-sinkage parameter. Therefore, for the validation of the bevameter developed in this study, a comparison with the existing literature [3], which performed a test with a pressure plate of the same size as Case # 1(D60, D100), was performed. As a result, the values were similar to existing literature [3], which confirmed that the equipment and test did not have errors. Table 4 lists the values from Bekker [3] and those measured in this study.

### 3.2. Shear Test

**Figure 13:**
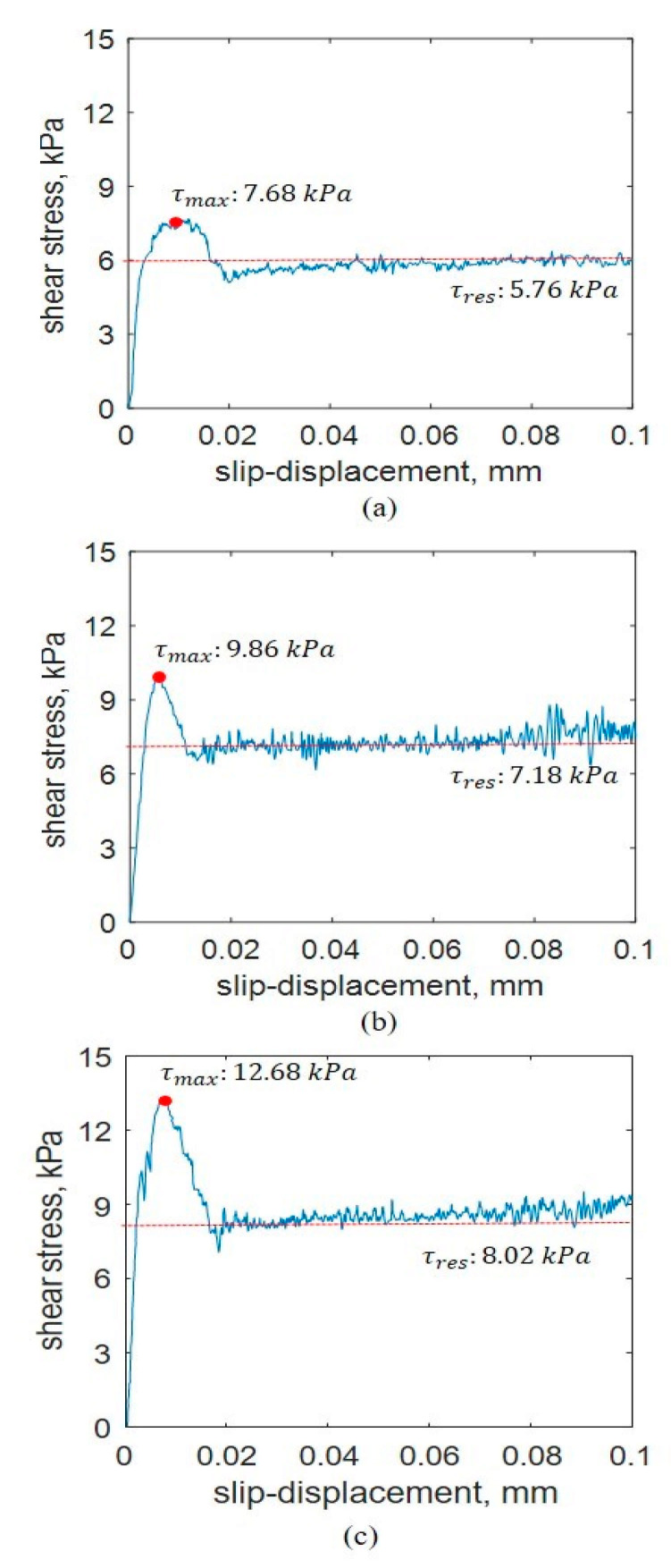
Shear stress–slip displacement curves at different normal pressures: (**a**) 14.04, (**b**) 19.89, and (**c**) 25.74 kPa.

In the shear test, the normal pressure was calculated by dividing the applied dead load (self-load of 176.58 N with weight) by the shear ring area (0.033537 m^2^), and a rotating velocity of 0.044 rad/s was applied for all the tests. Figure 13 shows the shear stress–slip displacement of the soil at each normal pressure. Table 5 lists the shear stress parameters at each normal pressure. Equation (3) [20] was used to derive the shear stress parameters because the shear stress–slip displacement relationship indicated soil softening behavior. Table 6 indicates that increasing the normal pressure increased the maximum shear stress, residual shear stress, and ratio of the residual shear stress to the maximum shear stress. The increases in the maximum shear stress and residual shear stress were attributed to the increase in the confining pressure applied to the soil as the normal pressure was increased. In addition, the increase in the maximum shear stress was greater than the increase in the residual shear stress.

**Figure 14:**
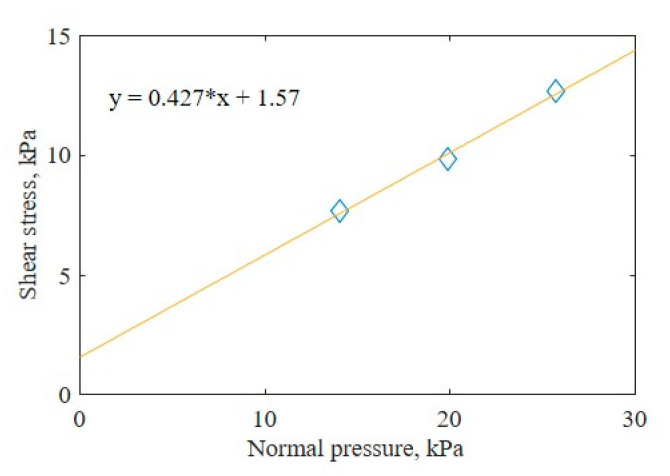
Results with the Mohr–Coulomb failure criterion.

Shear tests were conducted at different normal pressures to define the relationship between the shear stress and the normal pressure. The measurements and Mohr–Coulomb failure criterion were used to derive the cohesion and angle of internal friction of the sample soil. Figure 14 shows the result with the Mohr–Coulomb failure criterion. Table 7 compares the shear stress parameters of sand according to Bekker [3] and the values measured in this study. 

**Figure 15:**
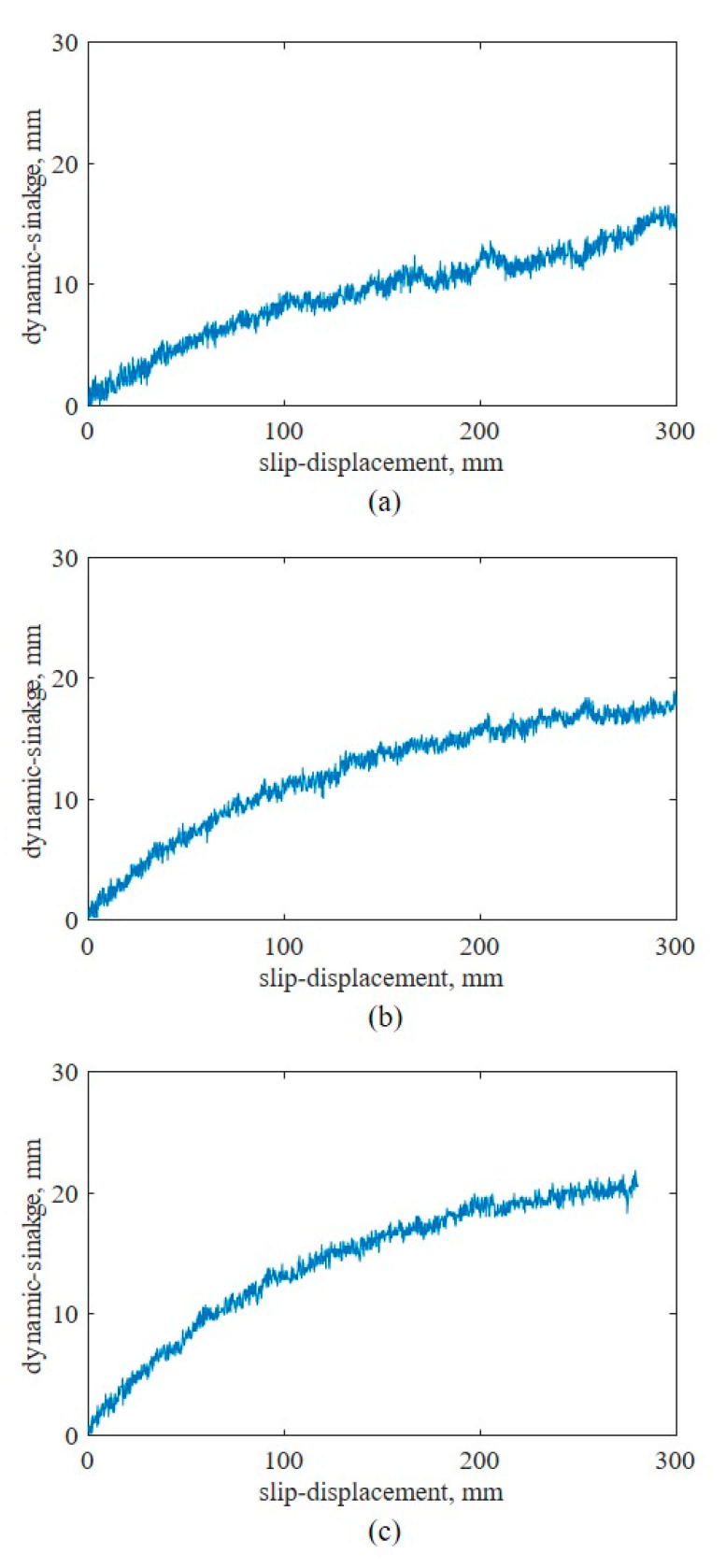
Slip sinkage–slip displacement curves at different normal pressures: (**a**) 14.04, (**b**) 19.89, and (**c**) 25.74 kPa.

The developed bevameter was designed to induce subsidence during shear by the addition of a spline to the shear test device. Therefore, the shear test could be used to determine not only the shear stress–slip displacement relationship but also the slip sinkage–slip displacement relationship. Figure 15 shows the slip sinkage–slip displacement relationship at each normal pressure. The slip sinkage also tended to increase in proportion with the slip displacement, but the slope of the increase gradually decreased. Because the initial slope of the slip sinkage tended to increase in proportion with the normal pressure, the slip sinkage was confirmed to be proportional to the normal pressure and slip displacement. However, at the maximum normal pressure of 25.74 kPa, the maximum length of the spline attached to the shear test device exceeded 20 mm before a slip displacement of 300 mm was achieved. This indicates that a longer spline is required for analysis of the slip sinkage–slip displacement relationship in weak soil.

## 4. Conclusions

In this study, a bevameter that can measure the mechanical properties of soil was developed. It can predict the thrust and motion resistance of an off-road vehicle on the soil surface, which are closely related to the driving performance. Tests were conducted to evaluate the developed equipment. The soil texture, moisture content, and bulk density of the sample soil were measured and confirmed to match those of sand. Furthermore, pressure–sinkage and shear tests were performed. For the pressure–sinkage test device, the test results confirmed that smaller plate size reduced the pressure–sinkage parameter *n*. In addition, the values derived in this study were compared with the pressure–sinkage parameters of sand reported by Bekker [3]. The results confirmed that the derived values were within the acceptable ranges for sand. For the shear test device, normal pressures were applied to the shear ring with a dead load, instead of hydraulic pressure, and a spline was installed on the shaft to decouple the normal pressure and torque of the shear ring. The spline rendered it possible to measure the slip sinkage using the slip displacement. Shear tests were conducted at different normal pressures, and the results showed that the slip sinkage increased in proportion with the normal pressure and slip displacement, but the slope gradually decreased. This study confirmed that sinkage could be predicted from the pressure–sinkage and shear stress-slip displacement relationship, rather than the pressure–sinkage relationship alone. This enables more accurate prediction of the sinkage and motion resistance, which tend to be underestimated. The shear-stress parameters measured using the developed bevameter were compared with those reported by Bekker [3] for sand and determined to be within acceptable ranges. The results establish that the developed bevameter can be used to predict the slip sinkage–slip displacement relationship in sand.

## Figures and Tables

**Table 1:** Specifications of the sensors in the bevameter.

Item	Model	Specifications
Load cell	YG38-500k	Rated capacity: 4905 N Rated output: 2 mV/V ± 0.1% Nonlinearity: 0.05%
LVDT	DWS-12R	Rated range: 1.2 m Nonlinearity: 0.25% Repeatability: 0.008%
Torque meter	YDRA-20 k	Rated capacity: 196 N∙m Rated output: 1.5 mV/V ± 0.1% Nonlinearity: 0.09% Repeatability: 0.09%
Rotary encoder	E100H 35-10000	Resolution: 10,000 Voltage output: Max. 1 µs Max. response frequency: 300 kHz
Laser displacement sensor	DT35-B15551	Rated range: 50 mm Resolution: 0.1 mm Repeatability: 0.5 mm Response time: 4.5 ms
Data acquisition device	DEWE-43A	Number of channels: 8 Sampling rate: 200 kS/s Noise floor: 107 dB @ ±10 V range Channel-to-channel phase mismatch: <0.1°@ 5kHz

**Table 2:** Physical properties of the sample soil.

Soil Texture	Sand
Moisture content, %	0.2
Bulk density, $g/{\mathrm{cm}}^{3}$	1.4

**Table 3:** Pressure–sinkage parameters for each case.

	Case #1 (D60, D100)	Case #2 (D40, D60)	Case #3 (D60, D80)	Case #4 (D80, D100)
$K_{c}$, $\mathrm{kN}/m^{n+1}$	3.76	−66.63	35.39	82.99
$K_{\emptyset }$, $\mathrm{kN}/m^{n+2}$	1979.17	4985.45	1584.61	394.56
n, dimensionless	1.07	0.97	0.97	1.09

**Table 4:** Comparison of the pressure–sinkage parameters of Bekker [3] and this study.

Soil Texture: Sand	Bekker [3]	This Study
$K_{c}$, $\mathrm{kN}/m^{n+1}$	0.99	3.76
$K_{\emptyset }$, $\mathrm{kN}/m^{n+2}$	1528.43	1979.17
n, dimensionless	1.10	1.07

**Table 5:** Normal pressure with a dead load.

Dead Load, kN	Normal Pressure, kPa
0.47	14.04
0.67	19.89
0.86	25.74

**Table 6:** Shear stress–slip displacement parameter at each normal pressure.

Normal Pressure	14.04 kPa	19.89 kPa	25.74 kPa
$\tau_{max},\;\mathrm{kPa}$	7.68	9.86	12.68
$\tau_{res},\;\mathrm{kPa}$	5.76	7.18	8.02
$K_{W},\;m$	0.015	0.007	0.009
$K_{r},\;\mathrm{dimensionless}$	0.749	0.728	0.632

**Table 7:** Shear stress parameters of Bekker (1969) and this study.

Soil Texture: Sand	Bekker (1969)	This Study
Cohesion c, kPa	1.04	1.57
Angle of internal friction ∅, °	28.00	23.12

## Data Availability

We are not able to make our data available to the public because of privacy constraints.
